# Initial Clinical Experience with a Modulated Holmium Laser Pulse—Moses Technology: Does It Enhance Laser Lithotripsy Efficacy?

**DOI:** 10.5041/RMMJ.10315

**Published:** 2017-10-16

**Authors:** Michael Mullerad, Jonatan R. A. Aguinaga, Tareq Aro, Alexander Kastin, Oleg Goldin, Alexander Kravtsov, Akram Assadi, Shadie Badaan, Gilad E. Amiel

**Affiliations:** Department of Urology, Rambam Health Care Campus, Haifa, Israel

**Keywords:** Holmium, laser, lithotripsy, stone, ureteroscopy

## Abstract

**Objective:**

The Lumenis^®^ High-power Holmium Laser (120H) has a unique modulated pulse mode, Moses™ technology. Moses technology modulates the laser pulse to separate the water (vapor bubble), then deliver the remaining energy through the bubble. Proprietary laser fibers were designed for the Moses technology. Our aim was to compare stone lithotripsy with and without the Moses technology.

**Methods:**

We designed a questionnaire for the urologist to fill immediately after each ureteroscopy in which the Lumenis 120H was used. We compared procedures with (*n*=23) and without (*n*=11) the use of Moses technology. Surgeons ranked the Moses technology in 23 procedures, in comparison to regular lithotripsy (worse, equivalent, better, much better). Laser working time and energy use were collected from the Lumenis 120H log.

**Results:**

During 4 months, five urologists used the Lumenis 120H in 34 ureteroscopy procedures (19 kidney stones, 15 ureteral stones; 22 procedures with a flexible ureteroscope, and 12 with a semi-rigid ureteroscope). Three urologists ranked Moses technology as much better or better in 17 procedures. In 2 cases, it was ranked equivalent, and in 4 cases ranking was not done. Overall, laser lithotripsy with Moses technology utilized laser energy in less time to achieve a satisfying stone fragmentation rate of 95.8 mm^3^/min versus 58.1 mm^3^/min, *P*=0.19. However, this did not reach statistical significance.

**Conclusion:**

The new Moses laser technology demonstrated good stone fragmentation capabilities when used in everyday clinical practice.

## INTRODUCTION

Modern renal stone surgery maximizes stone removal while minimizing invasiveness. Extracorporeal shockwave lithotripsy (ESWL) is the least invasive treatment with acceptable stone-free rates (70%–85%) and the standard treatment for small kidney stones (up to 2 cm).^1^ Percutaneous nephrolithotripsy (PCNL) is the gold standard for large and complex kidney stone disease (more than 2 cm).^1^,^2^

In the last two decades advances in technology made ureteroscopic access to kidney stones possible, and, as ureteroscopy made the visualization of the target stone feasible, a compatible flexible tool to fragment the stone had to be developed.

That came in the form of the pulsed holmium yttrium-aluminium-garnet (YAG) laser—a solid-state laser system that operates at a wavelength of 2140 nm. Holmium laser is absorbed by water, and therefore is absorbed superficially by tissue, allowing for cutting or ablation of strictures and tumors. The main use of the holmium YAG laser is for lithotripsy—urinary stone fragmentation.^3^ The holmium YAG laser has two mechanisms of action when fragmenting urinary stones: one produces a cavitation bubble that generates a weak shockwave. The other, a photothermal mechanism, requires direct absorption of laser energy. Following holmium laser targeting, the presence of glowing hot stone fragments suggests holmium laser lithotripsy utilizes a photothermal mechanism to cause stone vaporization.^4^

Stone movement caused by laser shockwave (proximal stone migration, i.e. retropulsion) is one disadvantage of laser lithotripsy.^5^,^6^ As stone evacuation capabilities by ureteroscopy are limited, the fragmentation of stones to multiple small stones is another disadvantage when high stone burden disease is treated by laser.^6^ Hence a dusting mode is used to evaporate the stone to dust. Due to lack of flexibility of the laser fiber itself, the ureteroscope may not be able to reach urinary stones in difficult anatomical positions (e.g. kidney lower calyx stones).

The new Lumenis High-power Holmium YAG laser (120H; Yokneam, Israel) enables the surgeon to control the pulse width, and it features a unique modulated pulse mode (Moses), designed to minimize stone retropulsion.^7^ Moses technology modulates the laser pulse to separate the water (vapor bubble), then deliver the remaining energy through the bubble.^7^ Proprietary laser fibers were designed by Lumenis for the Moses mode with a minimal diameter of 200 μm. Our aim in this study was to compare laser stone fragmentation with and without the Moses mode and proprietary laser fiber as experienced by urologists whose field of excellence is ureteroscopy laser lithotripsy.

## METHODS

We designed a questionnaire for the urologist to fill immediately after each ureteroscopy in which the Lumenis 120H was used. We compared procedures with and without the use of Moses technology. Over a period of four months the Lumenis 120H was evaluated in 36 procedures by five different urologists. We excluded two patients who were treated for upper tract transitional cell carcinoma; the rest of the patients had urinary stone disease.

In 23 procedures the Moses technology was used, while regular lithotripsy was used in 11 procedures. We used Moses laser fibers with a diameter of 200 μm, 365 μm, and 550 μm, and regular fibers of 200 μm and 365 μm. Surgeons ranked each of 23 procedures, in comparison to regular mode (worse, equivalent, better, much better). The operators were also asked about their subjective experience with the Moses technology capabilities in specific domains: stone fragmentation rate, ability to minimize stone retropulsion, fiber flexibility, and fiber durability (all on a scale of 1–4: 4, excellent; 3, good; 2, average; 1, poor). Laser working time and energy use were collected from the Lumenis 120H log for all 34 procedures. Stone sizes were taken from the preoperative computerized tomography (CT) scan. Stone volume and total stone burden were calculated by measuring the stones’ three dimensions in millimeters and then calculating the stone volume: length × width × height × π × 1/6.^8^,^9^

Clinical data were collected from the patients’ medical file. The study was approved by the institutional ethical committee.

## RESULTS

The comparison groups were similar in terms of demographics and by entry clinical data. Table 1 summarizes patient characteristics (age, gender, stone volume, stone CT attenuation in Hounsfield units) and demonstrates a similar distribution of patients in terms of stone size and attenuation.

**Table 1:** Patient Clinical Characteristics.

	Standard-Treated (*n*=11)	Moses-Treated (*n*=23)	*P*-value	Overall
Age, average (median), y	51.9±11.8 (51)	54.6±16.1 (58)	0.62	53.7±14.7 (54.5)
Gender, M/F	10/1	15/8	0.21	25/9
Total stone volume, median (25%–75%), mm^3^	422.5 (182.2–875.3)	781.9 (180.7–1691.3)	0.48	560.5 (192–1549.3)
NCCT (HU), median (25%–75%)	867 (502–1268)	901.5 (553.5–1085.0)	0.98	895 (550–1090)

NCCT, non-contrast CT; HU, Hounsfield Unit.

Stone locations were: the kidney in 19 patients, the ureter in 15 patients. In these procedures, a flexible ureteroscope was used in 22 cases and a semi-rigid ureteroscope in 12.

The Moses technology was used in 23 procedures, by three urologists. In all 23 procedures, the lithotripsy fragmentation rate was rated either excellent (15 cases) or good (8 cases). The domain of minimizing stone retropulsion was rated excellent in 8 procedures, good in 10, average in 2, and not rated in 3 cases. Moses fiber durability was rated excellent in 19 cases and good in 4. Fiber flexibility was rated excellent in 12 procedures, good in 4, average in 2, and not rated in 5. When overall lithotripsy efficacy of the Moses technology was compared to regular lithotripsy in these procedures, in 3 procedures the surgeon rated the Moses as much better, in 15 procedures it was ranked better, and in 2 procedures equivalent. In 3 procedures a comparison of the surgeon’s impression was not available.

The median calculated stone fragmentation rate for Moses and standard technology was 95.8 mm^3^/min versus 58.1 mm^3^/min, respectively (Table 2), thus supporting the surgeons’ subjective evaluation that Moses technology allows for larger stone volumes to be fragmented in a shorter time span; however, these results did not reach statistical significance (*P*=0.19).

**Table 2:** Median Stone Volume, Total Energy Used, Working Time, and Fragmentation Rate.

	Standard-Treated (*n*=11)	Moses-Treated (*n*=23)	*P*-value	Overall
Total stone volume, median (25%–75%), mm^3^	422.5 (182.2–875.3)	781.9 (180.7–1691.3)	0.48	560.5 (192–1549.3)
Energy use (kJ), median (25%–75%)	6.4 (2.6–11.9)	4.5 (1.6–16.0)	0.87	4.5 (1.6–12.9)
Laser working time (min), median (25%–75%)	10 (2.5–15.0)	6 (2.8–13.0)	0.46	8 (3–13)
Stone fragmentation rate, volume/working time (25%–75%), mm^3^/min	58.1 (30.8–102.4)	95.8 (51.5–177.4)	0.19	87.1 (32.4–130.1)

kJ, kilojoule; min, minute

## DISCUSSION

As new technologies have been developed in the field of minimally invasive lithotripsy, we notice a paradigm shift in stone disease clinical practice. Treatment using ESWL, considered to be the gold standard for small kidney and upper ureteral stones, is pushed aside by ureteroscopy as a treatment modality for kidney and upper ureteral stones (ureteroscopy for kidney stone often referred to as retrograde intrarenal surgery, RIRS).^1^,^2^,^10^ With a stone-free rate above 90% for a first ureteroscopic procedure compared to 67%–83% in ESWL,^11^–^13^ it is not surprising that patients and urologists turn to ureteroscopy as the first-line treatment when available. The development of both laser technology and ureteroscopic equipment allows the surgical treatment of kidney stones as big as 2 cm in diameter that were once treated only by PCNL.^1^,^2^ These new methods, completed with minimal complications, allow a much less invasive alternative to PCNL. Since ureteroscopy is already considered the standard of care for mid and lower ureteral stones, it is no wonder that this procedure is becoming more frequent in most endo-urology services.^2^

However successful, ureteroscopy is heavily dependent on technology. Another consideration is the cost of disposable equipment as well as reusable sensitive equipment (ureteroscope). The recent advances in digital imaging and the development of digital high-definition ureteroscopy improve the quality of imaging at the time of surgery. Advances in these technologies now allow for the first time a disposable flexible high-definition ureteroscope to be produced and competitively priced. With time and competition these technologies, now deemed too expensive in many health care systems across the globe, will become affordable.

The pulsed holmium YAG laser is the powerful tool that completes the ureteroscopy, allowing the cutting or ablation of strictures and tumors and the fragmenting of urinary stones.^3^

Stone retropulsion at time of laser lithotripsy as well as the fragmentation of stones to multiple small stones is one hurdle that laser lithotripsy technology needs to address. Another is the lack of flexibility of the laser fiber, hampering the ureteroscope’s reach of urinary stones in difficult anatomical positions.

Holmium laser lithotripters allow the operator to control the pulse energy and pulse frequency. Recently, newer models of holmium lasers enable the urologist to choose different pulse durations. In both settings the same amount of energy is delivered, but retropulsion is reduced when the energy is distributed on a longer pulse.^5^,^14^–^16^

Previous studies were focused on pulse amplitude and frequency for reducing the size of fragments. These studies demonstrated that a lower amplitude reduces retropulsion and fragment sizes.^6^

Elhilali et al. evaluated the Moses technology with an *in vitro* model as well as *in vivo* animal model.^7^ The *in vitro* model demonstrated pronounced reduction of retropulsion in the Moses mode during fragmentation setting (high energy, low frequency) as well as dusting mode (low energy, high frequency). Moses modes resulted in a significantly higher ablation volume when compared with the regular mode (160% higher; *P*<0.001). *In vivo* assessment also supported the reduction in retropulsion when treating stones in the porcine kidney.^7^

As the Lumenis 120H with the Moses technology is now approved for clinical use and commercially available, we sought to compare the surgeon operator experience with the Moses mode as well as the objective efficacy of this technology in fragmenting stones.

The subjective clinical evaluation of the Moses technology by three experienced urologists in everyday clinical practice was excellent on all domains tested. The operators experienced less stone retropulsion when working with the Moses technology. Domains linked to laser fiber quality (durability and flexibility) were good as well.

Comparing the urologist lithotripsy experience when operating with the Moses mode, nearly every time Moses mode lithotripsy was ranked better.

Objective data collected included treated stone burden volume, laser working time, and energy use, which allowed us to calculate the median stone volume fragmentation rate (volume of stone fragmented per unit time). The higher fragmentation rate with the Moses mode (95.8 mm^3^/min versus 58.1 mm^3^/min, Table 2) did not reach statistical significance (*P*=0.19), probably due to the small sample size. However, this trend gives some support to the surgeons’ subjective evaluation.

Our study is a non-randomized trial with a small study group and multiple operators, and as such is limited in the clinical outcome analysis it can offer. We were able to draw conclusions about subjective surgeon experience and objective stone fragmentation rate; however, we do not have sufficient follow-up and sample size to compare stone-free rate at this stage. Further studies assessing Moses technology impact on stone-free rate are warranted.

We conclude that in our experience in an everyday clinical practice our initial impression using Moses technology is that it allows a considerable reduction in stone retropulsion. Although not statistically significant, this study reports promising results that warrants further clinical studies in large and varied groups of patients.

## Abbreviations

CT: computerized tomography
ESWL: extracorporeal shockwave lithotripsy
laser: light amplification by stimulated emission of radiation
PCNL: percutaneous nephrolithotripsy
RIRS: retrograde intrarenal surgery
YAG: yttrium-aluminium-garnet.
